# Metabolite Profiling Reveals Abiotic Stress Tolerance in Tn5 Mutant of *Pseudomonas putida*

**DOI:** 10.1371/journal.pone.0113487

**Published:** 2015-01-28

**Authors:** Vasvi Chaudhry, Anil Bhatia, Santosh Kumar Bharti, Shashank Kumar Mishra, Puneet Singh Chauhan, Aradhana Mishra, Om Prakash Sidhu, Chandra Shekhar Nautiyal

**Affiliations:** Council of Scientific & Industrial Research-National Botanical Research Institute, Rana Pratap Marg, Lucknow, Uttar Pradesh, India; National Institute of Plant Genome Research, INDIA

## Abstract

*Pseudomonas* is an efficient plant growth–promoting rhizobacteria (PGPR); however, intolerance to drought and high temperature limit its application in agriculture as a bioinoculant. Transposon 5 (Tn5) mutagenesis was used to generate a stress tolerant mutant from a PGPR *Pseudomonas putida* NBRI1108 isolated from chickpea rhizosphere. A mutant NBRI1108T, selected after screening of nearly 10,000 transconjugants, exhibited significant tolerance towards high temperature and drought. Southern hybridization analysis of *Eco*RI and *Xho*I restricted genomic DNA of NBRI1108T confirmed that it had a single Tn5 insertion. The metabolic changes in the polar and non-polar extracts of NBRI1108 and NBRI1108T were examined using ^1^H, ^31^P nuclear magnetic resonance (NMR) spectroscopy and gas chromatography-mass spectrometry (GC-MS). Thirty six chemically diverse metabolites consisting of amino acids, fatty acids and phospholipids were identified and quantified. Insertion of Tn5 influenced amino acid and phospholipid metabolism and resulted in significantly higher concentration of aspartic acid, glutamic acid, glycinebetaine, glycerophosphatidylcholine (GPC) and putrescine in NBRI1108T as compared to that in NBRI1108. The concentration of glutamic acid, glycinebetaine and GPC increased by 34%, 95% and 100%, respectively in the NBRI1108T as compared to that in NBRI1108. High concentration of glycerophosphatidylethanolamine (GPE) and undetected GPC in NBRI1108 indicates that biosynthesis of GPE may have taken place via the methylation pathway of phospholipid biosynthesis. However, high GPC and low GPE concentration in NBRI1108T suggest that methylation pathway and phosphatidylcholine synthase (PCS) pathway of phospholipid biosynthesis are being followed in the NBRI1108T. Application of multivariate principal component analysis (PCA) on the quantified metabolites revealed clear variations in NBRI1108 and NBRI1108T in polar and non-polar metabolites. Identification of abiotic stress tolerant metabolites from the NBRI1108T suggest that Tn5 mutagenesis enhanced tolerance towards high temperature and drought. Tolerance to drought was further confirmed in greenhouse experiments with maize as host plant, where NBRI1108T showed relatively high biomass under drought conditions.

## Introduction

PGPR from genus *Pseudomonas* are known to colonize in the rhizosphere of a wide range of plants growing in diverse habitats and stimulate their growth through direct or indirect mechanisms. Plants are exposed to a wide range of abiotic stresses including edaphic stress. Efficient commercial symbionts like *Pseudomonas* are expected to optimize plant growth in crops cultivated in difficult conditions, however, *Pseudomonas* is intolerant to high temperature and drought, which is a limitation [1,2].

Advances in recombinant DNA technology and classic strain engineering approaches have enabled modifications in organisms. Tn5 is a versatile tool that can generate mutants with altered functions. Its transposition in bacterial DNA is random and results in single-site, non-leaky, polar mutations with a selectable phenotype. There are reports where Tn5 mutants have exhibited better activity as compared to NBRI1108 strain. NBRI1108T of *P. putida* showed enhanced temperature tolerance and toluene-resistance [3]. NBRI1108T of *Azospirillum lipoferum* has been reported to significantly alter indole acetic acid (IAA) production [4], while in another study, Tn5 NBRI1108T expressed over four-fold more bacteroid cytochrome-c oxidase in *Bradyrhizobium japonicum* [5].

State of the art spectroscopy tools are being successfully used to decipher metabolic changes in engineered organisms. ^1^H NMR spectroscopy has assisted in metabolic identification and quantification of metabolites in complex biological mixtures and plant extracts [6]. ^31^P NMR spectroscopy has been used for quantification of cellular and plasma membrane phospholipids and many other phosphorous containing metabolites from biological samples [7,8]. GC–MS is a robust and a widely used technique that combines high sensitivity and specificity for specific analyte classes using chemical derivatization [9]. In view of the potential of *Pseudomonas* species for application in drought prone and degraded sites to enhance productivity in tropical conditions, an investigation was undertaken to enhance drought tolerance performance in *P. putida* by using Tn5 mutagenesis as a tool to generate NBRI1108Ts with single, stable and random integration in the genome of the isolated strain. The main objective of the present study was to investigate the metabolic alterations, related to drought and temperature tolerance and their possible pathways, caused by Tn5 insertion in *P. putida* by using a combination of sophisticated tools like ^1^H, ^31^P NMR spectroscopy and GC-MS. The study also attempts to elucidate the phenotypic and genetic attributes of the microbe, understand the process by which NBRI1108T imparts stress tolerance ability to the microbe, and identify biomarkers for abiotic stress tolerance generated by Tn5 insertion.

## Materials and Methods

### Bacterial strain and generation of Tn5 mutagenesis

The plant growth promoting rhizobacterial strain of *P. putida* (NBRI1108) was earlier isolated from chickpea (*Cicer arietinum* L. cv. Radhey) roots grown under rain-fed conditions at Dholpur district, Rajasthan, India. The strain has been deposited in the Microbial Type Culture Collection, Chandigarh, India under the accession number MTCC5279 [10]. We have already reported its plant growth promotional attributes such as presence of auxin and the ability to solubilize phosphate [10,11]. Tn5 was introduced in NBRI1108 by conjugation with *Escherichia coli* WA803⁄pGS9 as per an earlier described protocol [12]. Transconjugants were selected on M9 minimal basal salt medium consisting of (per liter): Na_2_HPO_4_, 22 g; KH_2_PO_4_, 1 g; NaCl, 1 g; NH_4_Cl, 2 g; sucrose, 4 g; MgSO_4_, 1 mM; CaCl_2_, 0.25 mM and biotin, 1 mg with 1.5% (w/v) agar with kanamycin (50 μg/mL) and ampicillin (50 μg/mL) to avoid growth of auxotrophs. Genomic DNAs from NBRI1108 and NBRI1108T strains were isolated and digested with *Eco*RI or *Xho*I restriction endonuclease. The fragments were separated by agarose gel electrophoresis. The presence of single insertion of Tn5 was detected by Southern hybridization analysis of nitrocellulose blots of the agarose restriction patterns by using a ^32^P-labeled Tn5 probe, consisting of 2.3-and 2.4-kb internal fragments of Tn5 generated after its complete *Xho*I digestion following earlier reported methods [13–15].

### Selection strategy for stress tolerant NBRI1108T

The drought and temperature tolerant Tn5 mutant NBRI1108T was selected after screening various transconjugants of NBRI1108. Stress tolerance of transconjugants was tested by growing them on nutrient broth (NB) containing 45% polyethylene glycol 6000 for drought, and at 40°C for high temperature tolerance. The inoculum of transconjugants consisting of ~7 log_10_ CFU/mL was incubated in a refrigerated incubator shaker at 180 rpm. Viable cell counts of NBRI1108 and NBRI1108T were determined by harvesting samples at different intervals of time. The CFU/mL of NBRI1108 and NBRI1108T at each time point was calculated in triplicate using serial dilutions plating of each sample on nutrient agar (NA) plates and incubated at 30°C.

### Sample preparation for metabolite measurements

The NBRI1108 and NBRI1108T were grown in NB medium to attain the logarithmic growth phase (~ 9 Log_10_ CFU/mL); harvested in chilled tubes by centrifugation at 5000 g for 10 minutes and washed twice with cold phosphate buffered saline (PBS; NaCl, 137 mM; KCl, 2.7 mM; Na_2_HPO_4_, 10 mM; KH_2_PO_4_, 2 mM; pH 7.4). Metabolites from bacterial cells were extracted in a binary mixture of chloroform-methanol (2:1 v/v) using ultrasonicator (Sonics, Newtown, CT, USA) following Folch’s method [16]. Polar and non-polar metabolites from chloroform-methanolic extract were separated by centrifugation of the samples at 2400 rpm for 20 minutes at 4°C. The polar phase was lyophilized and prepared for NMR analysis as described by Kruger *et al*. [17]. The phase containing non-polar metabolites was concentrated under reduced pressure till removal of the solvent was complete. Samples were stored at −20°C till further analysis. The metabolite profiling of polar and non-polar extracts was performed using GC-MS, ^1^H and ^31^P NMR spectroscopy.

### GC-MS analysis

GC-MS analysis of lipid content was performed using Thermo Trace GC Ultra coupled with Thermo fisher DSQ II Mass Spectrometers. Chromatographic separations of metabolites were carried out on 30 m x 0.25 mm Thermo TR50 column (polysiloxane column coated with 50% methyl and 50% phenyl groups). X-calibur software was used to process the chromatography and mass spectrometry data. The GC oven temperature was maintained at 70°C for 5 minutes, then gradually increased by 5°C per minute till it reached 290°C and this temperature was maintained for 5 minutes. The sample was injected in the split mode at a splitting ratio of 1:16. Helium was used as a carrier gas and set at a constant flow rate of 1 ml/min. The mass selective detector was run in the electron impact (EI) mode, with electron energy of 70 eV. The resulting GC-MS profile was analyzed using WILLY and NIST mass spectral library by matching the chromatogram with commercially available standards and further confirmed by trimethylsilyl (TMS) derivatization. A freely available mass spectral deconvolution algorithm (Automated Mass Spectral Deconvolution and Identification System, AMDIS32) was used for processing the multiple datasets. Approximately 5 mg of the sample was suspended in 40 μL of methoxylamine hydrochloride in GC grade pyridine (20 mg/ml) for preparing volatile TMS derivative of the sample. The mixture was shaken for 2 hours at 37°C in a temperature controlled vortex followed by the addition of 70 μL of the N-methyl-N-(trimethylsilyl-trifluoroacetamide (MSTFA), and continuous shaking for another 30 minutes. All GC-MS data were further normalized using nonadecanoic acid as an internal standard [18]. However, concentration of non-polar metabolites was calculated on percent peak area basis.

### ^1^H NMR analysis

The ^1^H NMR spectra of polar extracts were recorded at 300K on Bruker Biospin Avance-III 800 MHz NMR (Bruker GmbH, Germany) spectrometer equipped with a triple resonance cryoprobe. 1D and 2D NMR spectral analyses of aqueous extracts were carried out by dissolving the samples in 500 μL D_2_O in 5mm NMR tubes. A sealed capillary of 20 μL of deuterium oxide containing 0.03% (w/v) sodium salt of trimethylsilyl propionic acid (TSP) was used for quantitative estimation of metabolites; it also served as an internal lock. ^1^H NMR spectra with water suppression was obtained using one-dimensional single pulse with 65,536 time domain data points, spectral width of 12,019.23 Hz, a relaxation delay of 5.0 seconds, acquisition time of 2.72 seconds, 64 number of scans with 8 dummy scans, free-induction decay resolution 0.36 Hz, a constant receiver gain of 32 with offset frequency set 3771.16 Hz. The ^1^H NMR spectra of plant extracts were manually phased and automated baseline was corrected using TOPSPIN 2.1 (Bruker Analytik, Rheinstetten, Germany). The ^1^H NMR spectra were referenced to the methyl resonance of alanine at 1.48 ppm. For confirm the assignments, two-dimensional ^1^H–^1^Hcorrelated spectroscopy (COSY) and ^1^H–^13^C heteronuclear single quantum coherence (HSQC) were performed using Bruker’s standard pulse program library. The assignments were further confirmed by comparing them with the existing literature values.

### ^31^P NMR analysis

Samples for ^31^P NMR analysis were prepared by mixing 10 mg of dried non-polar extracts of NBRI1108 and NBRI1108T strains, separately in 500 μL binary mixture of CDCl_3_-MeOD (2:1 v/v). ^31^P NMR spectra were recorded on Bruker Biospin Avance-I 400 MHz NMR (Bruker Biospin, Switzerland) spectrometer operating at a proton frequency of 400.12 (Hz) using 5mm broad band inverse probe equipped with z-gradient accessories. A WILMAD co-axial insert containing sodium salt of MDP (methylene diphosphonic acid) in D_2_O was used for the quantitative estimation of metabolites and that also served as chemical shift reference. ^31^P NMR spectra with proton decoupling were acquired with the following experimental parameters: spectral width of 19379.84 Hz, time domain data points of 32K, effective 30° flip angle, relaxation delay of 5.0 seconds, acquisition time of 0.85 seconds, 4096 number of scan(s) with 8 dummy scan(s).

### In-plant-drought tolerance assay

Comparative performance of NBRI1108 and NBRI1108T under drought stress was evaluated under greenhouse conditions using maize as host plant grown in earthen pots containing sterilized soil. Bacterial inoculum of NBRI1108 and NBRI1108T for maize seeds was prepared by suspending 48 h grown cultures from NA plates at 28°C in 10 ml of 0.85% saline Milli-Q water (MQW), containing about 8 log_10_ CFU/ml. Surface-sterilized maize seeds were soaked in the bacterial suspension for 4 h at 28°C on a reciprocal shaker at 100 rpm. Control seeds (non-bacterized) were soaked in 0.85% saline MQW washed from uninoculated NA plates. For drought stress 3 weeks old plants were subjected to progressive drought by no further addition of water, whereas normal plants were regularly watered to maintain the 20% moisture. Plants were harvested after 4 weeks and data on root length, shoot length and plant dry biomass were recorded.

### Statistical analysis

Statistical analysis of plant biomass and relative percentage peak area of GC-MS data were carried out by using SPSS 11.5 version. Quantified NMR data of NBRI1108 and NBRI1108T strains of *P. putida* were pareto-scaled using Microsoft Excel 2007 (Microsoft Corporation, USA). These were further imported to the Unscrambler X Software package (Version 10.2 CAMO, USA) for multivariate PCA. The *p*-values < 0.05 were used to calculate significance.

## Results and Discussion

**Figure 1:**
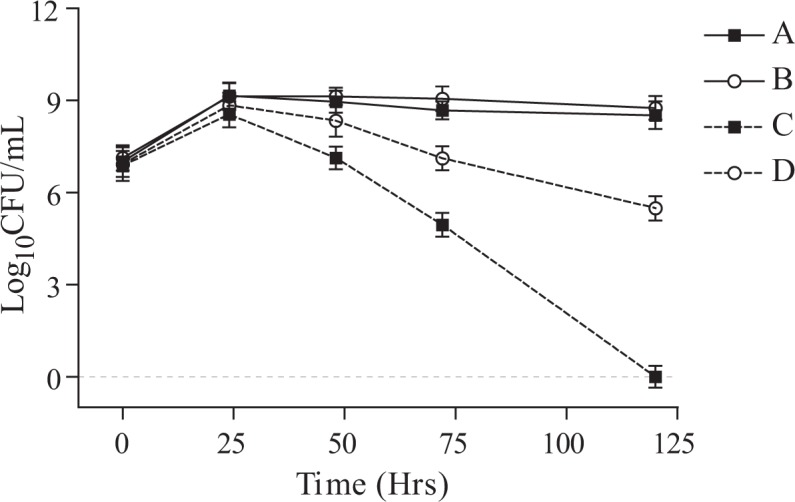
Effect of temperature on the survival of *P. putida* NBRI1108 and NBRI1108T. Treatments are designated as (A) NBRI1108 at 28 ± 2°C, (B) NBRI1108T at 28 ± 2°C, (C) NBRI1108 at 40°C and (D) NBRI1108T at 40°C. Values are the mean ± SE of three samples.

**Figure 2:**
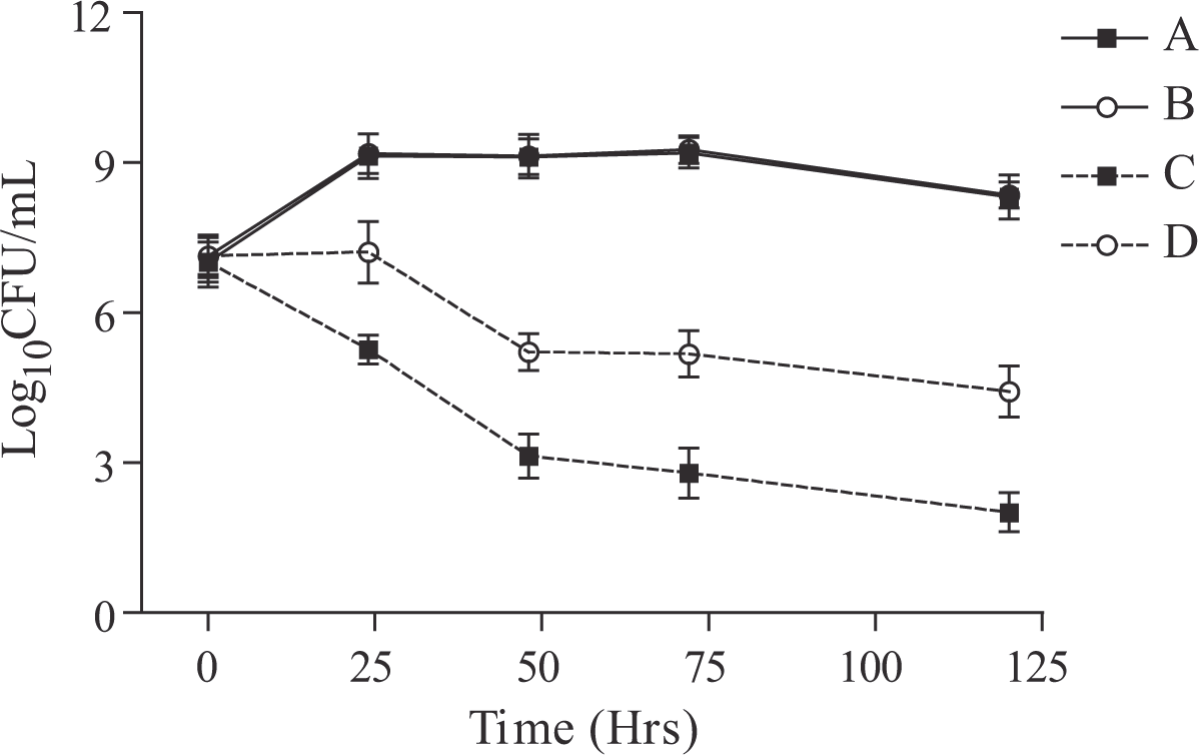
Effect of drought on the survival of *P. putida* NBRI1108 and NBRI1108T. Treatments are designated as (A) NBRI1108 without PEG6000, (B) NBRI1108T without PEG6000, (C) NBRI1108 with 45% PEG6000 and (D) NBRI1108T with 45% PEG6000. Values are the mean ± SE of three samples.

**Figure 3:**
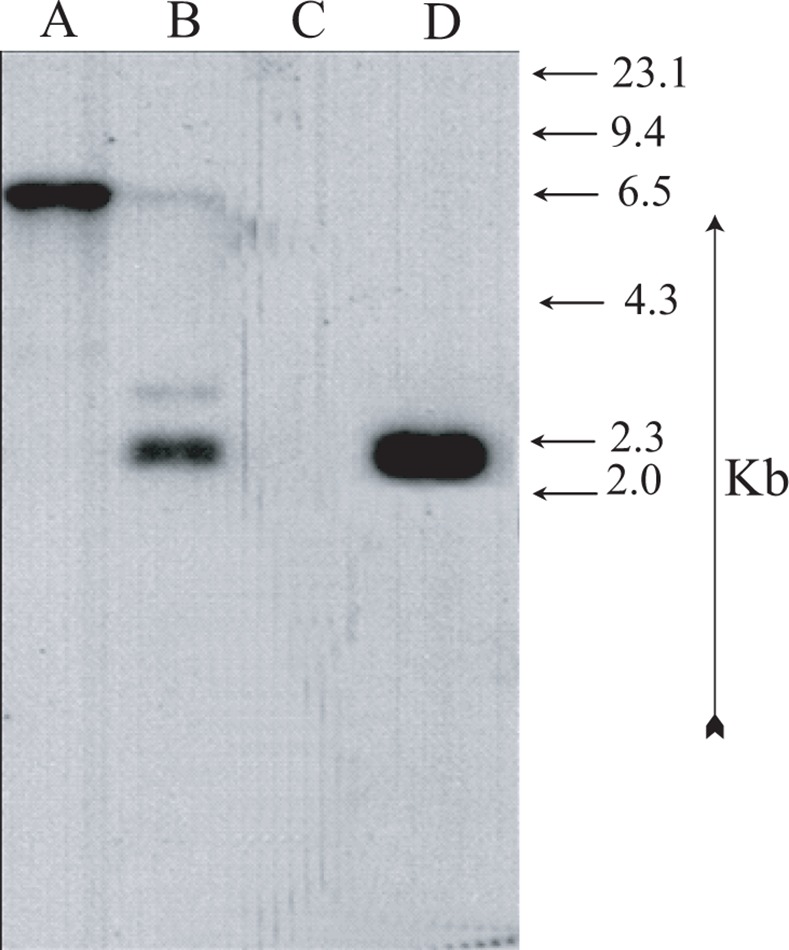
Southern hybridization analysis of total genomic DNA of *P. putida* NBRI1108 and NBRI1108T. (A) *Eco*RI digested NBRI1108T (B) *Xho*I digested NBRI1108T (C) *Eco*RI digested NBRI1108 and (D) *E*. *coli* WA803 ⁄pGS9 DNA as positive control for the ^32^P-labelled Tn5 probe as described in materials and methods. Molecular size values in the margins are in kilobases.

The present study was undertaken to develop stress tolerant *Pseudomonas* NBRI1108T by using Tn5 insertion and comparing the metabolic alterations responsible for stress amelioration in the host plant in NBRI1108 under stressed conditions. Tn5 mutant, NBRI1108T of *P. putida* NBRI1108, was selected after screening nearly 10000 transconjugants of the NBRI1108. The NBRI1108T showed enhanced tolerance to drought and high temperature and it was able to grow optimally in NB medium amended with 45% PEG 6000 and NB medium exposed to 40°C after 5 days of incubation as shown in Figs. 1 and 2. Single band in *Eco*RI and 4 bands in *Xho*I digested lanes indicate single insertion of Tn5 in NBRI1108T, whereas no band was observed in *Eco*RI digested NBRI1108 lane in NBRI1108 by using Southern hybridization (Fig. 3). The stability and single insertion of Tn5 in NBRI1108T was in agreement that done in the previous studies [12–15].

Metabolite profiling of polar and non-polar extracts of NBRI1108 and NBRI1108T was investigated. Thirty six, polar and nonpolar, chemically diverse intracellular metabolites consisting of amino acids, dicarboxylic acids, fatty acids, glycerols, phospholipids and metabolites of energy metabolism such as ATP and UTP were identified using ^1^H, ^31^P NMR spectroscopy (Table 1) and GC-MS (Table 2).

**Table 1:** Variability in ^1^H and ^31^P NMR identified metabolites of *P. putida* NBRI1108 and NBRI1108T.

Metabolite	^1^H Chem. Shift	Concentration (μg/g fresh weight, n = 3)		*p*-value
		NBRI1108	NBRI1108T	
Isoleucine	0.92 (t), 0.99 (d)	14.11 ± 1.21	14.04 ± 1.58	NS
Valine	1.04 (d), 1.01 (d)	29.24 ± 2.84	27.65 ± 2.20	NS
Lactate	1.33 (d), 4.12 (q)	76.90 ± 14.44	55.02 ± 7.77	NS
Alanine	1.48 (d), 3.78 (q)	67.60 ± 13.41	69.53 ± 12.63	NS
Putrescine	1.77, 3.06	131.69 ± 42.57	355.77 ± 66.21	0.008
Acetate	1.91 (s)	16.58 ± 5.59	7.03 ± 2.94	NS
Glutaric Acid	2.18 (t), 1.78 (m)	6.18 ± 0.97	35.21 ± 6.14	0.013
GABA	1.94 (m), 2.31 (t), 3.02 (t)	79.61 ± 15.86	18.02 ± 5.18	0.003
Glutamic Acid	2.07 (m), 2.35 (t), 3.74 (m)	476.94 ± 82.22	721.30 ± 55.49	0.013
Succinate	2.41(s)	81.16 ± 20.70	87.01 ± 4.93	NS
Aspartic acid	2.65–2.78 (m), 3.90 (dd)	61.65 ± 8.59	156.50 ± 10.41	0.001
Glycinebetaine	3.27 (s), 3.91 (s)	33.71 ± 4.65	701.29 ± 52.02	0.002
Glycine	3.56 (s)	81.28 ± 14.85	88.91 ± 6.39	NS
N-acetylglucosamine	4.66 (d), 5.21 (d)	54.34 ± 12.31	0.00 ± 0.00	0.002
UDP-glucuronate	5.56(d)	31.97 ± 6.67	0.00 ± 0.00	0.01
Fumaric acid	6.52 (s)	2.46 ± 0.74	0.00 ± 0.00	0.03
Tyrosine	6.89 (d), 7.19 (d)	12.82 ± 2.21	10.03 ± 1.10	NS
Phenylalanine	7.32 (m), 7.40 (m)	17.62 ± 3.28	17.57 ± 2.75	NS
UDP/UTP	6.0 (d), 7.95 (d)	129.78 ± 32.78	155.70 ± 17.56	NS
ATP	8.26 (s), 8.60 (s)	242.70 ± 43.56	291.97 ± 42.84	NS
NADP	8.11 (s), 8.42 (s), 8.83 (d), 9.13 (d), 9.33 (s)	280.99 ± 50.15	411.87 ± 64.43	0.03
Arginine	3.74, 3.25, 1.69	NQ	NQ	−
Taurine	3.25, 3.41	NQ	NQ	−
GPC	3.12 (s)*	ND	2667.86 ± 176.54	0.004
GPE	3.8 (s)*	12471.36 ± 438.03	1216.43 ± 146.35	0.008
iP	4.42 (s)*	852.79 ± 52.68	658.73 ± 132.03	0.08

Values are means of 3 replicates ±SE

* = ^31^P Chemical Shift

p-values ≤ 0.05 were considered as significant

NS: Not Significant

iP: inorganic phosphate

NQ: Not Quantified

**Table 2:** Qualitative and quantitative assignments of CHCl_3_: MeOH (2:1) soluble metabolites identified by GC-MS in the extract of *P. putida* NBRI1108 and NBRI1108T.

Compound Name	tR (min)	Mass Fragmentation	Concentration (%) (N = 3)		*p*-value
			NBRI1108	NBRI1108T	
Glycerol (3TMS)	12.39	*m/z* 308(M^+^), 293(M^+^-CH_3_), 205, 147, 133,73*	0.32 ± 0.06	0.83 ± 0.03	0.005
Diethylene glycol (2TMS)	13.44	*m/z* 235(M^+^- CH_3_), 191, 147, 117,73	0.12 ± 0.03	0.18 ± 0.04	0.13^NS^
Lauric acid (1TMS)	24.04	*m/z* 272(M^+^), 257(M^+^-CH_3_), 145, 132, 117, 55,73	0.40 ± 0.04	0.57 ± 0.05	0.014
α-Glycerophosphate (4 TMS)	26.24	*m/z* 460(M^+^), 445, 389, 373, 249, 191, 147, 103,73	5.35 ± 0.26	3.24 ± 0.18	0.001
Myristic acid (1TMS)	28.22	*m/z* 300(M^+^), 285 (M^+^-Me), 147,73	1.08 ± 0.06	1.40 ± 0.11	0.022
Palmitic acid (1TMS)	32.07	*m/z* 328(M^+^), 313(M^+^-CH_3_), 285,73	24.27 ± 2.12	20.61 ± 3.16	0.19^NS^
Margaric acid (1TMS)	33.88	*m/z* 370(M^+^), 355(M^+^-CH_3_), 262,73	ND	0.52 ± 0.02	0.001
Palmitelaidic acid (1TMS)	34.02	*m/z* 326(M^+^), 311(M^+^-CH_3_), 236,73	1.95 ± 0.05	0.73 ± 0.02	0.001
Stearic acid (1TMS)	35.60	*m/z* 356(M^+^), 341(M^+^-CH_3_), 328, 297,73	16.79 ± 2.10	22.68 ± 2.61	0.06^NS^
Arachidic acid (1TMS)	38.90	*m/z* 382(M^+^), 367(M^+^-CH_3_),73	1.40 ± 0.10	1.94 ± 0.03	0.012

*p*-values ≤ 0.05 are considered as significant. Mean ± SD. ND = Not Detected, NS = Not Significant, 73* = *m/z* 73 in all TMS derivatized metabolites has Me_3_Si peak of 100% relative abundance.

### Metabolic profiling by ^1^H NMR and ^31^P NMR

Twenty three chemically diverse metabolites consisting of amino acids, dicarboxylic acids, trimethylglycine and metabolites of energy metabolism such as ATP, UTP, UDP and NADP were identified through ^1^H NMR (Table 1). Assignments of polar metabolites were carried out by comparing the ^1^H NMR spectra of reference compounds with the existing literature values [18]. Reference compounds in the Biological Magnetic Resonance Data Bank (BMRB) were also used for characterizing the metabolites [19]. In the high-field region of the ^1^H NMR spectra (0.8–4.7 ppm) of aqueous extracts, the most abundant peaks corresponded to isoleucine, leucine, valine, ethanol, lactate, alanine, putrescine, acetate, glutaric acid, glutamic acid, GABA, succinic acid, aspartic acid, lysine, taurine, arginine, betaine and glycine (S1 Fig.). In the down-field region (5.6–10.0 ppm) signals were assigned for UDP-glucuronate, UDP/UTP, NADP/ATP, fumaric acid, tyrosine and phenylalanine (S2 Fig.). Assignments were confirmed by COSY spectrum (S3 Fig.).

^1^H NMR spectral complexity (overlapping signals) did not allow quantification of all the metabolites. However, 21 of these were quantified by integrating the distinct characteristic signals of each metabolite with respect to the intensity of the nine protons of TSP (in D_2_O, 0.375%, w/v) on dry weight basis of bacterial cells [18]. Concentration of metabolites in NBRI1108 and NBRI1108T (Table 1). The NBRI1108T showed significantly higher concentration of putrescine, glutaric acid, glutamic acid, aspartic acid and glycinebetaine as compared to that in NBRI1108. Some of the metabolites such as isoleucine, valine, alanine, phenylalanine and tyrosine were detected in nearly equal amounts in both the strains. However, concentration of lactate, acetate, GABA, NADP, n-acetylglucosamine and UDP-glucuronate, fumaric acid and ATP was relatively high in NBRI1108 as compared to that in NBRI1108T (Table 1). The NBRI1108T showed significantly higher concentration of aspartic acid, glutamic acid, glutaric acid, glycinebetaine and putrescine as compared to that in NBRI1108. Tn5 insertion increased the concenteration aspartic and glutamic acid by 60% and 34%, respectively in NBRI1108T as compared to that in NBRI1108. Bacteria have been reported to accumulate high levels of aspartic acid and glutamic acid under osmotic stress conditions [20,21]. Tn5 mutagenesis increased putrescine by 63% in Tn5 NBRI1108T as compared to that in NBRI1108 thus indicating higher thermoprotectant properties of the former. Long-chain and branched polyamines, plays an important role in stabilizing DNAs and RNAs at high temperatures in thermophilic bacteria [22]. PGPR have been reported to produce osmoprotectants under stress conditions which modulate their cytoplasmic osmolarity [23]. Increase in glycinebetaine by more than 20-fold in NBRI1108T as compared to that in NBRI1108 indicates higher osmoprotectant and thermoprotectant capability of the former. Higher intracellular concentration of glycinebetaine has been reported to play an important role in osmoregulation in bacteria [24]. Synthesis of metabolites like aspartic acid, glutamic acid, glycinebetaine and putrescine in NBRI1108T indicate their possible role in imparting osmoregulatory properties to the mutant fir it to survive in drought conditions.

Presence of GPC, GPE and an inorganic phosphate (Pi) were identified from ^31^P NMR spectra with GPC at 3.12 ppm, GPE at 3.8 ppm, and Pi at 4.42 ppm (S4 Fig.). Assignments of metabolites were carried out with support from the existing literature values [25].

Assignments of GPC and GPE were further confirmed by ^1^H NMR experiments of organic layers of NBRI1108 and NBRI1108T (S5 Fig.). GPC was not detected in NBRI1108 while it was present in high concentration (2.6 mg/g) in NBRI1108T (Table 1). Phospholipids are important for maintaining membrane fluidity that get altered with variations in the temperature and other environmental conditions [26]. GPC and GPE have been reported to be present in abundence in eukaryotic cells and many bacteria [27]. However, GPE decreased significantly in NBRI1108T as compared to that in NBRI1108. It did not follow the same trend as GPC. Similarly, there was an insignificant change in the synthesis of Pi in the two strains. GPC is an important membrane forming phospholipid, which accumulates in bacteria [28] and yeast cells in response to an increase in temperature [29] or osmotic stress [30]. Earlier, workers have reported a significant variation in phospholipids in solvent-tolerant strains of *P. putida* [31]. Accumulation of GPC in NBRI1108T shows the ability of the mutant to tolerate stress conditions like drought and temperature.

### Metabolic profiling by GC-MS

Ten metabolites consisting of saturated and unsaturated fatty acids and glycerol were identified in chloroform extracts of both NBRI1108 and NBRI1108T (Table 2) using REPLIB, WILEY and NIST mass spectral library and reference compounds. There was a significant increase in glycerol content in the NBRI1108T (Table 2). A relatively high percent peak area was also observed for lauric acid, myristic acid, margaric acid and arachidic acid in the NBRI1108T as compared to that in NBRI1108. Palmitic acid was the dominant fatty acid with a concentration of 24.3% and 20.6% in NBRI1108 and NBRI1108T, respectively. Bacteria have been reported to precisely adjust their membrane lipid composition by modifying the fatty acids and altering the structures of pre-existing phospholipids in diverse environments [32]. Tn5 insertion increased glycerol content by 61% in NBRI1108T as compared to that in NBRI1108 suggesting alteration in metabolism in *P. putida*. A significantly higher glycerol content in Tn5 NBRI1108T as compared to that in NBRI1108 shows the ability of NBRI1108T to tolerate osmotic stress. Earlier workers have investigated the role of sugar alcohols in osmotic stress adaptation and reported that *Saccharomyces cerevisiae* cells accumulate glycerol, to compensate for differences between the extracellular and intracellular water potential in response to high external osmotic environment [33]. NBRI1108T enhanced components of stress protectants especially, aspartic acid, GPC, glutamic acid, glycine betaine and putrescine in *P. putida*, suggesting adaptation of mutated strain towards tolerance of abiotic stress.

### Principal component analysis

**Figure 4:**
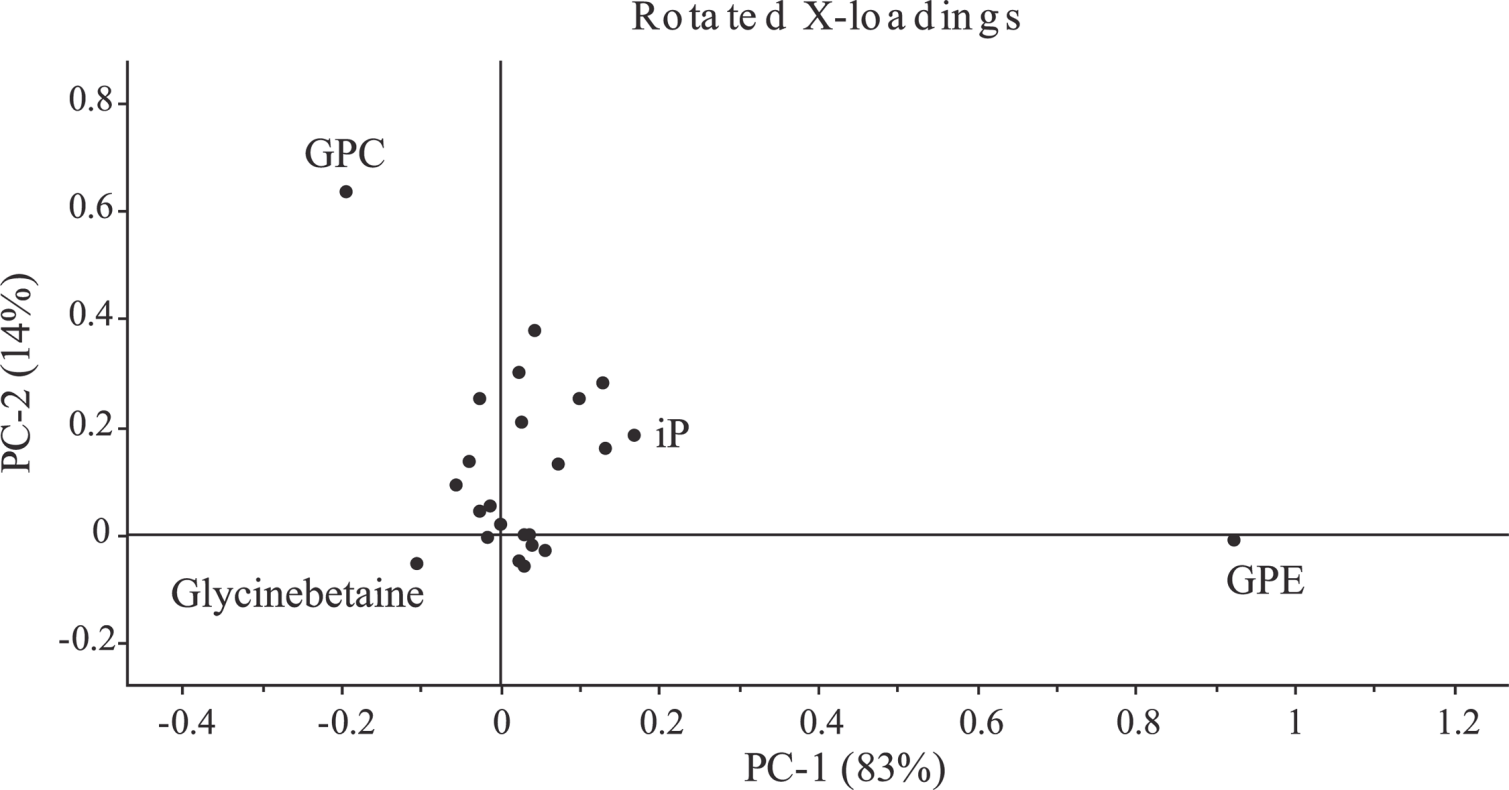
Principal component analysis of quantified polar and nonpolar metabolites of *P. putida* NBRI1108 and NBRI1108T.

The multivariate PCA was applied to quantified polar and nonpolar metabolites of NBRI1108 and NBRI1108T of *P. putida*. PC1 vs PC2 score plot with its corresponding scattered loadings is shown in Fig. 4. A total 97% explained variance could be described with these two principal components. GPE and GPC were found to be the major confounders for group separation between NBRI1108 and NBRI1108T. They assisted in figuring out the dominant phospholipid (GPC and GPE) biosynthetic pathway (S6 Fig.).

### Analysis of plant growth promotion under drought stress

Greenhouse trials were carried out in normal and drought conditions to evaluate the effect of inoculation of NBRI1108 and NBRI1108T in maize plants to ameliorate the effect of drought stress on the plants. The results revealed that the NBRI1108T inoculation has enhanced root and shoot growth by 38% and 16% and 40% total dry biomass as compared to non-inoculated control under drought stress condition (Table 3). Increase in growth and biomass because of inoculating the maize plants grown in drought conditions with NBRI1108T shows the potential of Tn5 mutant to withstand stress.

**Table 3:** Effect of inoculation of *P. putida* NBRI1108 and NBRI1108T on the growth of maize plant in drought stress.

Parameters	Control			Drought stress		
	Non- inoculated	Inoculated with		Non- inoculated	Inoculated with	
		NBRI1108	NBRI1108T		NBRI1108	NBRI1108T
Root length (cm)	40.88 ±4.10^c^	49.78 ±5.83^b^	49.60 ±4.97^b^	34.33 ±3.27^d^	42.01 ±4.36^c^	55.93 ±4.84^a^
Shoot length (cm)	50.64 ±6.42^b^	70.83 ±1.82^a^	69.98 ±8.12^a^	43.50 ±2.46^c^	50.07 ±2.12^b^	51.85 ±2.18^b^
Root dry weight (g)	0.37 ±0.10^d^	0.79 ±0.25^a^	0.79 ±0.25^a^	0.27 ±0.10^e^	0.47 ±0.17^c^	0.65 ±0.17^b^
Shoot dry weight (g)	0.79 ±0.16^c^	2.06 ±0.53^a^	2.52 ±1.26^a^	0.52 ±0.17^d^	0.78 ±0.28^c^	1.43 ±0.36^b^
Total Biomass (g)	1.16±0.26^c^	2.84±0.78 ^a^	3.31±1.51^a^	0.80±0.27^d^	1.25±0.45^c^	2.08±0.53^b^

Values are mean of 3 replicates ±SE; Different letters indicate significant differences among treatments as revealed by Duncan’s multiple range test at p ˂ 0.05.

Remarkable differences between NBRI1108T and NBRI1108 suggest that changes in metabolic pathway result in synthesis of metabolites crucial for providing drought tolerance to the microbe. Comparative metabolic profiling of NBRI1108T and NBRI1108 revealed that Tn5 mutagenesis altered amino acid, phospholipids and polyol metabolism resulting in significantly enhanced concentration of aspartic acid, glutamic acid, glycinebetaine, GPC and putrescine. Putrescine is biosynthesized by ornithine decarboxylase (ODC) and arginine decarboxylase (ADC) pathways [34]. Presence of putrescine, glutamic acid and arginine in aqueous extracts of both NBRI1108 and NBRI1108T indicates that putrescine may have been synthesized via the arginine decarboxylase (ADC) pathway in *P. putida*. Arginine is involved in a variety of functions including intracellular signaling, stress resistance and RNA as well as protein synthesis in bacteria [35,36]. Arginine is derived from glutamate, a primary substrate of putrescine biosynthesis [37]. Hence higher occurrence of these metabolites suggests that NBRI1108T may have relatively high stress resistance as compared to NBRI1108. The hypothesis was further supported by the fact that there was a several-fold increase in glycinebetaine in NBRI1108T as compared to that in NBRI1108. Higher intracellular concentration of glycinebetaine plays an important role in osmoprotection and thermoprotection.

Altered metabolism of phospholipids in NBRI1108T, when investigated using ^31^P NMR, revealed a very high concentration of GPC as compared to that in NBRI1108. Qualitative difference in phospholipids between the two strains suggests different routes of phospholipid biosynthesis (S6 Fig.). High concentration (12.4 mg) of GPE and undetected GPC in NBRI1108 indicate that biosynthesis of GPE may have taken place via the methylation pathway of phospholipid biosynthesis. However, high GPC concentration and low GPE in NBRI1108T suggest that both methylation pathway and PCS pathway of phospholipid biosynthesis are being followed in NBRI1108T. A metabolic linkage map represents routes of polyamine and phospholipid metabolic pathways in NBRI1108 and NBRI1108T of *P. putida* (S6 Fig.).

Tn5 insertion altered phospholipid metabolism resulting in accumulation of higher concentration of GPC, an important component of cell membrane as compared to NBRI1108 of *P. putida*. Earlier, biosynthesis of phosphatidylcholine has been reported to occur via the PCS pathway in *P. putida* ATCC 12633 [38,39]. Both, methylation and PCS pathways for phosphatidylcholine biosynthesis have been reported in bacteria [39]. Option of both pathways in the NBRI1108T assisted changes in polyamine, trimethylamine and phospholipid biosynthesis resulted in accumulation of glycinebetaine, putrescine and GPC. These metabolites, in turn, assisted the *P. putida* NBRI1108T in enhancing tolerance to temperature and drought stresses.

## Conclusion

Experimental data and evidences suggest that Tn5 mutation significantly altered the metabolism in *P. putida* (NBRI1108). The combined application of ^1^H, ^31^P NMR and GC-MS platforms have complemented each other in generating detailed information about the metabolites which would not have been possible by using the individual methods separately. NBRI1108T had significantly altered amino acid and phspholipid metabolism that resulted in increase in concentration of aspartic acid, glycerol, GPC, glutamic acid, glycinebetaine and putrescine. Significant variations in the levels of GPE and GPC in the two strains suggest that mutation may have activated the methylation pathway of phosphatidylcholine biosynthesis. Increase in betaine and GPC levels consequently led NBRI1108T to tolerate drought and temperature toa greater extent. Application of multivariate PCA to the quantified metabolites revealed a clear separation between NBRI1108 and NBRI1108T strains of *P. putida*. Drought and temperature stress tolerance conferred by NBRI1108T in *P. putida* represents a novel, compelling approach towards improving plant productivity with mutant symbiont. The study suggests that the Tn5 generated mutant NBRI1108T can efficiently be used in drought stress condition for higher productivity.

## Supporting Information

S1 Fig High field region of ^1^H NMR spectra of polar extract. NBRI1108 (A) and NBRI1108T (B) strains of *P. putida*.
(EPS)

S2 Fig Low field region of ^1^H NMR spectra of polar extract. NBRI1108 (A) and NBRI1108T (B) strains of *P. putida*.
(EPS)

S3 Fig ^1^H–^1^H COSY spectrum of aqueous fraction along with the assignment of the metabolites (0.75–4.7ppm). (*Ile*u isoleucine, *Leu* leucine, *Val* valine, *Lys* lysine, *Gln* glutamine, *Arg* arginine, *Ala* alanine, *Lac* lactate, *Asp* aspartic acid, *GABA* γ-aminobutyric acid, *Cho* choline, *Tau* taurine, *Bet* betaine, *GA* glutaric acid, *EtOH* ethanol, *UK* unknown).
(EPS)

S4 Fig Stack-plot of ^31^P NMR spectra. *P. putida* NBRI1108 (A) and NBRI1108T (B).
(EPS)

S5 Fig Proton NMR spectra of lipids extract of *P. putida* cells showing detection of GPC and GPE. NBRI1108 (A) and NBRI1108T (B).
(EPS)

S6 Fig Simplified metabolic map displaying biosynthetic pathways for PC in *P. putida*. Metabolites denoted with star have shown significant changes in mutant.
(EPS)

## References

[1] Kloepper JW, Leong J, Teintze M, Schroth MN (1980) Enhanced plant growth by siderophores produced by plant growth-promoting rhizobacteria. Nature 286: 885–886.

[2] Srivastava S, Yadav A, Seem K, Mishra S, Chaudhary V, et al (2008) Effect of high temperature on *Pseudomonas putida* NBRI0987 biofilm formation and expression of stress sigma factor RpoS. Curr Microbiol 56: 453–457. 10.1007/s00284-008-9105-0 18219523

[3] Fukumori F, Hirayama H, Takami H, Inoue A, Horikoshi K (1998) Isolation and transposon mutagenesis of a *Pseudomonas putida* KT2442 toluene-resistant variant: involvement of an efflux system in solvent resistance. Extremophiles 2: 395–400. 9827328 10.1007/s007920050084

[4] Abdel-Salam M, Klingmüller W (1987) Transposon Tn5 mutagenesis in *Azospirillum lipoferum*: isolation of indole acetic acid mutants. Mol Gen Genet 210: 165–170.

[5] O'Brian MR, Kirshbom PM, Maier RJ (1987) Tn5-induced cytochrome mutants of *Bradyrhizobium japonicum*: effects of the mutations on cells grown symbiotically and in culture. J Bacteriol 169: 1089–1094. 3029019 10.1128/jb.169.3.1089-1094.1987 PMC211904

[6] Viant MR (2008) Recent developments in environmental metabolomics. Mol Biosyst 4: 980–986. 10.1039/b805354e 19082136

[7] Meneses P, Glonek T (1988) High resolution 31P NMR of extracted phospholipids. J Lipid Res 29: 679–689. 3411242

[8] Wright LC, Nouri-Sorkhabi MH, May GL, Danckwerts LS, Kuchel PW, et al (1997) Changes in cellular and plasma membrane phospholipid composition after lipopolysaccharide stimulation of human neutrophils, studied by 31P NMR. Eur J Biochem 243: 328–335. 9030756 10.1111/j.1432-1033.1997.0328a.x

[9] Styczynski MP, Moxley JF, Tong LV, Walther JL, Jensen KL, et al (2007) Systematic identification of conserved metabolites in GC/MS data for metabolomics and biomarker discovery. Anal Chem 79: 966–973. 17263323 10.1021/ac0614846

[10] Srivastava S, Chaudhry V, Mishra A, Chauhan PS, Rehman A, et al (2012) Gene expression profiling through microarray analysis in Arabidopsis thaliana colonized by *Pseudomonas putida* MTCC5279, a plant growth promoting rhizobacterium. Plant Signal Behav 7: 235–245. 10.4161/psb.18957 22353860 PMC3405686

[11] Chaudhry V, Asif MH, Bag S, Goel R, Mantri SS, et al (2013) Draft Genome Sequence of *Pseudomonas putida* Strain MTCC5279. Genome Announc 1: 1–2. 10.1128/genomeA.00560-13 PMC3731845 23908291

[12] Nautiyal CS, van Berkum P, Sadowsky MJ, Keister DL (1989) Cytochrome mutants of bradyrhizobium induced by transposon tn5. Plant Physiol 90: 553–559. 16666807 10.1104/pp.90.2.553 PMC1061760

[13] Chauhan PS, Nautiyal CS (2010) The purB gene controls rhizosphere colonization by Pantoea agglomerans. Lett Appl Microbiol 50: 205–210. 10.1111/j.1472-765X.2009.02779.x 20002573

[14] Rehman A, Nautiyal CS (2002) Effect of drought on the growth and survival of the stress-tolerant bacterium Rhizobium sp. NBRI2505 sesbania and its drought-sensitive transposon Tn5 mutant. Curr Microbiol 45: 368–377. 12232669 10.1007/s00284-002-3770-1

[15] Mishra S, Mishra A, Chauhan PS, Mishra SK, Kumari M, et al (2012) *Pseudomonas putida* NBRIC19 dihydrolipoamide succinyltransferase (SucB) gene controls degradation of toxic allelochemicals produced by Parthenium hysterophorus. J Appl Microbiol 112: 793–808. 10.1111/j.1365-2672.2012.05256.x 22324517

[16] Folch J, Lees M, Sloane Stanley GH (1957) A simple method for the isolation and purification of total lipides from animal tissues. J Biol Chem 226: 497–509. 13428781

[17] Kruger NJ, Troncoso-Ponce MA, Ratcliffe RG (2008) ^1^H NMR metabolite fingerprinting and metabolomic analysis of perchloric acid extracts from plant tissues. Nat Protocols 3: 1001–1012. 10.1038/nprot.2008.64 18536647

[18] Bhatia A, Bharti SK, Tewari SK, Sidhu OP, Roy R (2013) Metabolic profiling for studying chemotype variations in Withania somnifera (L.) Dunal fruits using GC-MS and NMR spectroscopy. Phytochemistry 93: 105–115. 10.1016/j.phytochem.2013.03.013 23578960

[19] Markley JL, Anderson ME, Cui Q, Eghbalnia HR, Lewis IA, et al. (2007) New bioinformatics resources for metabolomics. Pac Symp Biocomput: 157–168. 17990489

[20] Botsford JL, Lewis TA (1990) Osmoregulation in Rhizobium meliloti: Production of glutamic acid in response to osmotic stress. Appl Environ Microbiol 56: 488–494. 16348124 10.1128/aem.56.2.488-494.1990 PMC183366

[21] Canamas TP, Vinas I, Usall J, Magan N, Morello JR, et al (2007) Relative importance of amino acids, glycine-betaine and ectoine synthesis in the biocontrol agent Pantoea agglomerans CPA-2 in response to osmotic, acidic and heat stress. Lett Appl Microbiol 45: 6–12. 17594453 10.1111/j.1472-765X.2007.02156.x

[22] Oshima T (2007) Unique polyamines produced by an extreme thermophile, Thermus thermophilus. Amino Acids 33: 367–372. 17429571 10.1007/s00726-007-0526-z

[23] Talibart R, Jebbar M, Gouesbet G, Himdikabbab S, Wroblewski H, et al (1994) Osmoadaptation in Rhizobia—Ectoine-induced salt tolerance. J Bacteriol 176: 5210–5217. 8071195 10.1128/jb.176.17.5210-5217.1994 PMC196703

[24] Canovas D, Vargas C, Kneip S, Moron MJ, Ventosa A, et al (2000) Genes for the synthesis of the osmoprotectant glycine betaine from choline in the moderately halophilic bacterium Halomonas elongata DSM 3043. Microbiology 146: 455–463. 10708384 10.1099/00221287-146-2-455

[25] Kriat M, Vion-Dury J, Confort-Gouny S, Favre R, Viout P, et al (1993) Analysis of plasma lipids by NMR spectroscopy: application to modifications induced by malignant tumors. J Lipid Res 34: 1009–1019. 8354948

[26] Falcone DL, Ogas JP, Somerville CR (2004) Regulation of membrane fatty acid composition by temperature in mutants of Arabidopsis with alterations in membrane lipid composition. BMC Plant Biol 4: 17 15377388 10.1186/1471-2229-4-17 PMC524174

[27] Dowhan W (1997) Molecular basis for membrane phospholipid diversity: why are there so many lipids? Annu Rev Biochem 66: 199–232. 9242906 10.1146/annurev.biochem.66.1.199

[28] Medeot DB, Bueno MA, Dardanelli MS, de Lema MG (2007) Adaptational changes in lipids of Bradyrhizobium SEMIA 6144 nodulating peanut as a response to growth temperature and salinity. Curr Microbiol 54: 31–35. 17171469 10.1007/s00284-006-0233-0

[29] Dowd SR, Bier ME, Patton-Vogt JL (2001) Turnover of phosphatidylcholine in Saccharomyces cerevisiae. The role of the CDP-choline pathway. J Biol Chem 276: 3756–3763. 11078727 10.1074/jbc.M003694200

[30] Kiewietdejonge A, Pitts M, Cabuhat L, Sherman C, Kladwang W, et al (2006) Hypersaline stress induces the turnover of phosphatidylcholine and results in the synthesis of the renal osmoprotectant glycerophosphocholine in Saccharomyces cerevisiae. FEMS Yeast Res 6: 205–217. 16487344 10.1111/j.1567-1364.2006.00030.x

[31] Pinkart HC, White DC (1997) Phospholipid biosynthesis and solvent tolerance in *Pseudomonas putida* strains. J Bacteriol 179: 4219–4226. 9209036 10.1128/jb.179.13.4219-4226.1997 PMC179242

[32] Zhang Y-M, Rock CO (2008) Membrane lipid homeostasis in bacteria. Nat Rev Micro 6: 222–233. 10.1038/nrmicro1839 18264115

[33] Shen B, Hohmann S, Jensen RG, Bohnert a H (1999) Roles of sugar alcohols in osmotic stress adaptation. Replacement of glycerol by mannitol and sorbitol in yeast. Plant Physiol 121: 45–52. 10482659 10.1104/pp.121.1.45 PMC59388

[34] Nakada Y, Itoh Y (2003) Identification of the putrescine biosynthetic genes in Pseudomonas aeruginosa and characterization of agmatine deiminase and N-carbamoylputrescine amidohydrolase of the arginine decarboxylase pathway. Microbiology 149: 707–714. 12634339 10.1099/mic.0.26009-0

[35] Chattopadhyay MK, Tabor CW, Tabor H (2003) Polyamines protect Escherichia coli cells from the toxic effect of oxygen. Proc Natl Acad Sci U S A 100: 2261–2265. 12591940 10.1073/pnas.2627990100 PMC151328

[36] Wortham BW, Oliveira MA, Fetherston JD, Perry RD (2010) Polyamines are required for the expression of key Hms proteins important for Yersinia pestis biofilm formation. Environ Microbiol 12: 2034–2047. 10.1111/j.1462-2920.2010.02219.x 20406298 PMC3039482

[37] Page AF, Minocha R, Minocha SC (2012) Living with high putrescine: expression of ornithine and arginine biosynthetic pathway genes in high and low putrescine producing poplar cells. Amino Acids 42: 295–308. 10.1007/s00726-010-0807-9 21082203

[38] Boeris PS, Lucchesi GI (2012) The phosphatidylcholine synthase of *Pseudomonas putida* A ATCC 12633 is responsible for the synthesis of phosphatidylcholine, which acts as a temporary reservoir for Al^3+^ . Microbiology 158: 1249–1257. 10.1099/mic.0.054072-0 22343357

[39] Martínez-Morales F, Schobert M, López-Lara IM, Geiger O (2003) Pathways for phosphatidylcholine biosynthesis in bacteria. Microbiology 149: 3461–3471. 14663079 10.1099/mic.0.26522-0

